# Primary skeletal muscle diffuse large B cell lymphoma: A case report and review of the literature

**DOI:** 10.3892/ol.2015.3505

**Published:** 2015-07-17

**Authors:** LIJUAN ZHANG, QUANDE LIN, LINA ZHANG, LIHUA DONG, YUFU LI

**Affiliations:** 1 Transplantation Department of Internal Medicine, 302 Hospital of the Chinese People's Liberation Army, Beijing 100039, P.R. China; 2 Department of Hematology, Affiliated Cancer Hospital of Zhengzhou University, Zhengzhou, Henan 450008, P.R. China

**Keywords:** extranodal lymphoma, diffuse large B cell lymphoma, skeletal muscle, therapy

## Abstract

The occurrence of primary extranodal non-Hodgkin's lymphoma (NHL) of soft tissue is rare, particularly in skeletal muscle. The present study describes a case of diffuse large B cell lymphoma of the right lower extremity and provides a detailed review of the literature associated with this disorder, with the aim of improving the future diagnosis and therapy of extranodal NHL. The present case report was of a 76-year-old woman who presented with a right thigh and calf mass. In view of the tumor's location and the patient's age, soft tissue tumors were considered to be soft tissue sarcoma. Imaging scans were performed to determine the location and size of the tumor, followed by a biopsy of the muscle. Histopathological examination then yielded a diagnosis of diffuse large B cell lymphoma. The patient then underwent 4 cycles of chemotherapy. There was evident relief of pain and swelling in the right extremity; however, positron emission tomography/computed tomography (PET/CT) determined insufficient treatment efficacy. Chemotherapy was adjusted for 2 cycles; however, the patient suffered an aggravation of edema, so a different chemotherapy regimen of bleomycin, cytarabine, vincristine, cyclosphamide and dexamethasone (BCOAD) was performed for a further 2 cycles. The edema was alleviated and magnetic resonance imaging revealed shrinkage of the lower limb mass and the right thigh mass was undetectable. In conclusion, the present case report demonstrated that PET/CT may help determine the efficacy of chemotherapy treatment and that the BCOAD chemotherapy regimen may be more effective than standard treatments in certain cases.

## Introduction

Diffuse large B cell lymphoma (DLBCL) is the most prevalent form of non-Hodgkin's lymphoma (NHL) and has a high incidence rate among elderly people (1,2). The extranodal type of NHL is identified in 20–30% of patients (3). DLBCL often presents in extranodal sites, including the testis, skin, lung, bone and central nervous system, as well as the respiratory and gastrointestinal tracts (4). Primary skeletal muscle lymphoma is exceptionally rare form of DLBCL, particularly in the thigh and calf areas. The main symptoms of the disease typically include presence of a mass, pain and swelling (5). Imaging is conducted in order to establish a diagnosis, and the most common imaging techniques used include positron emission tomography/computed tomography (PET/CT) and magnetic resonance imaging (MRI). The present study reports a case of primary skeletal muscle lymphoma and provides a detailed review of the literature associated with this disorder.

## Case report

In March 2013, a 76-year-old female patient was admitted to the Affiliated Cancer Hospital of Zhengzhou University (Zhengzhou, China) and presented with a 1-year history of right foot numbness, which gradually worsened. In January 2013, the patient began experiencing pain and swelling in the right thigh and calf. However, the patient denied suffering from typical ‘B’ symptoms, including fever, night sweats and weight loss, and had no history of tobacco smoking, alcohol consumption or drug use.

Patient examination revealed a palpable firm mass of 54×48 mm in the right thigh and calf muscle, as well as several 10–28 mm firm, tender and mobile nodules distributed over the patients right groin and popliteal fossa. However, there was no evidence of hepatosplenomegaly. In addition, the patient had an Eastern Cooperative Oncology Group performance score of 2 (http://ecog-acrin.org/resources/ecog-performance-status).

**Figure 1.**
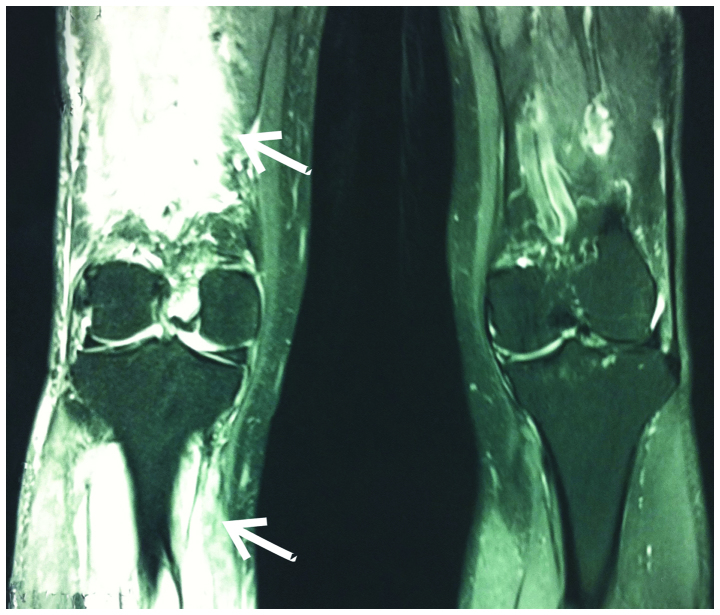
Features of the enhanced T1-weighted magnetic resonance imaging scans. Coronal section revealed a bulky uniform distension of skeletal muscles, which was hyperintense relative to normal muscle.

**Figure 2.**
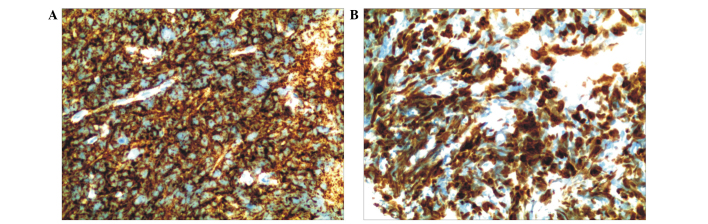
Immunohistochemistry of the patient. Immunohistochemical detection of (A) CD20 and (B) Ki-67 (magnification, ×200).

The blood test results were as follows: Hemoglobin, 10.5 g/dl (normal range, 12–16 g/dl); white cell count, 5.89×10^9^/l; platelets, 268×10^9^/l; lactate dehydrogenase, 466 U/l (normal range, 109–245 U/l); and β2-microglobulin, 3.81 mg/l (normal range, 0.8–2.2 mg/l). Serological tests for hepatitis B and C virus as well as human immunodeficiency virus were negative. MRI with enhanced T1-weighted sequence revealed a hyperintense mass in the lower tibia and upper fibula (Fig. 1), 90×54×48 mm in size. Enlarged lymph nodes were observed in the right popliteal, right inguinal and right iliac artery, 20–40 mm in size. CT scans revealed enlarged lymph nodes in the mediastinum, while the bilateral pulmonary region was normal. Bone marrow aspiration results were normal. A muscle biopsy was performed and the histological analysis revealed a diagnosis of primary skeletal muscle NHL DLBCL. Immunostaining of the tumoral cells demonstrated positive staining for CD20 and CD79α, which is typical of cells with B phenotype (Fig. 2A). Malignant cells were strongly positive for Ki-67 (70%) (Fig. 2B). In addition, CD3, CD43, epithelial membrane antigen, CD56 and synaptophysin were negative in the malignant cells. The patient was diagnosed as DLBCL stage IV, with an international prognostic index score of 4, indicating high risk (6).

**Figure 3.**
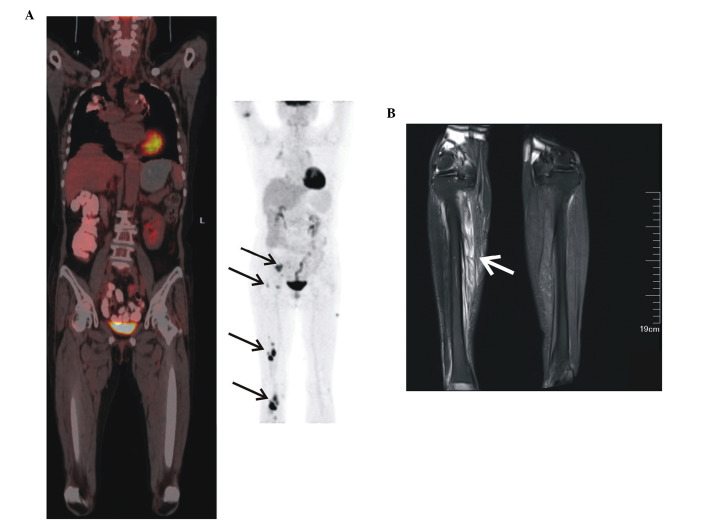
Imaging of the patient following chemotherapy. (A) FDG positron emission tomography/computerized tomography of the muscle following 4 cycles of chemotherapy demonstrated striking FDG uptake involving the skeletal muscles of the right thigh and calf muscle as well as the right iliac artery and right inguinal lymph nodes (arrows). (B) Coronal enhanced T1-weighted magnetic resonance imaging of the muscle following 8 cycles of chemotherapy. Coronal section revealed a diffuse, relatively homogenous hyperintense mass (arrow) in right calf muscles. FDG, fluorodeoxyglucose.

The patient received four cycles (21 days each) of R-CHOP chemotherapy, which involved administration of the following: 375 mg/m^2^ rituximab over 6 h on day −1; 750 mg/m^2^ cyclophosphamide, 2 mg vincristine and 50 mg/m^2^ doxorubicin (intravenous) on day 1; and 40 mg/m^2^ prednisolone (orally) on days 1–5, every 21-day cycle. Chemotherapy was administered intravenously once a month for a total of four cycles. The patient suffered neutropenia (absolute neutrophil count, <0.5×10^9^/l) and infectious complication during the first cycle. The patient was then treated with a modified R-Mini-CHOP (day 1, 600 mg/m^2^ cyclophosphamide, 2 mg/m^2^ vindesine and 375 mg/m^2^ rituximab; day 2, 50 mg/m^2^ epirubicin; and days 1–5, 40 mg/m^2^ prednisone) for the remaining three cycles. There was evident relief of pain and swelling in the right extremity. PET/CT imaging with 18F-fluorodeoxyglucose revealed a reduced fluorodeoxyglucose (FDG) uptake in the tumor and lymph nodes. However, in addition to the right thigh and calf muscle, FDG uptake persisted in the right iliac artery lymph nodes and right inguinal lymph nodes (Fig. 3A). PET/CT was used to evaluate the efficacy of the treatment, which revealed a stabilization of the disease. The patient was further treated with chemotherapy as follows: Day 1, 375 mg/m^2^ rituximab; day 3/7, 1000 mg/m^2^ gemcitabine; day 3–7, 15 mg/m^2^ cisplatin; and day 3–7, 9 mg dexamethasone (R-GDP) every 30 days for a total of 2 cycles. In October 2013 (2 months after chemotherapy), the patient suffered a gradual aggravation of edema, swelling and pain in the right lower limb. Doppler ultrasound results revealed an enlarged hypoechoic mass in the right calf, 72×43 mm in size. Subsequently, an adjusted chemotherapy regimen of bleomycin, cytarabine, vincristine, cyclosphamide and dexamethasone (BCOAD) regimen was administrated for 2 cycles. Subsequently, the patient received two cycles of R-BCOAD chemotherapy, which involved administration of the following: 375 mg/m^2^ rituximab over 6 h on day −1; 2.5 mg/m^2^ bleomycin, 0.5 mg vincristine and 10 mg/m^2^ cytarabine continuous infusion over 24 h on days 1–4; 650 mg/m^2^ cyclophosphamide on day 5; and 10 mg/m^2^ prednisolone (orally) on days 1–5, every 21-day cycle. This chemotherapy was administered through a continuous intravenous infusion of bleomycin, cytarabine and vincristine over 96 h. The edema was completely alleviated and the mass in the right lower limb began to shrink. MRI results revealed a reduction in tumor size in the lower limb and the mass in the right thigh became undetectable (Fig. 3B). The patient received a local radiation therapy; however, the effect of the treatment was poor and the mass gradually increased. The patient succumbed to the disease on July 2, 2014. Written informed consent was obtained from the patient's family prior to the publication of the present study.

## Discussion

Primary skeletal muscle lymphoma was first reported by Kandel *et al* (7) in 1984 and has since been reported to account for ~0.5% of extranodal lymphomas (8), with an incidence rate of <0.1% in all lymphoma of the extremities (9). Therefore, primary skeletal muscle NHL of diffuse large B cell immunophenotype is exceptionally rare. This disease may occur through one of the three ways: As disseminated disease via the hematogenous or lymphatic pathways; as an extension from adjacent organs, such as bones or lymph nodes; or very rarely, as primary extranodal disease (10).

The common clinical symptoms of primary skeletal muscle lymphoma are usually associated with muscle swelling, limb pain and edema, without any sign of heat and redness (5); in addition, this disease may occur as isolated lesions (11). The clinical features of the extranodal lymphoma include pain and tenderness, lymphadenopathy, ipsilateral extremity swelling and elevated lactate dehydrogenase, which therefore aid the diagnosis the primary skeletal muscle lymphoma (12). Furthermore, primary skeletal muscle lymphoma exhibits certain distinctive MRI features, which allow it to be differentiated from other types of soft-tissue tumors: On T1-weighted images, an increased signal intensity is commonly observed compared with normal muscle; and on T2-weighted images, intermediate signal intensity is observed compared with fat (11,13). In addition, on contrast-enhanced MRI, skeletal muscle lymphoma demonstrates homogeneous diffuse enhancement (14). CT scans may reveal muscle swelling and also serve as a tool to guide biopsy. With the development of technology, the clinical application of PET/CT has become increasingly important for lymphoma diagnosis and tumor staging (15). In the present study, PET/CT was employed in order to assess the efficacy of the chemotherapy treatments. However, imaging features of lymphoma in skeletal muscle are nonspecific and it may be difficult to distinguish lymphoma from other diseases, such as primary soft tissue, sarcoma, metastases, trauma or myositis (11). Therefore, biopsy and pathological evaluation are essential for the diagnosis of primary skeletal lymphoma (16). The present case report illustrated that MRI and CT provided the basis for diagnosis and that the diagnosis was confirmed through biopsy histopathology and immunohistochemistry.

The treatment of primary skeletal muscle lymphoma relies predominantly on the type of lymphoma. The prognosis of primary skeletal muscle lymphoma is poor compared with that of lymph node lymphoma, especially at stages III–IV. Therefore, selecting the most effective treatment regimen is essential. The present study reported a case of DLBCL, the standard treatment for which is R-CHOP (17–21).

The combination of chemotherapy and radiotherapy significantly was reported to increase disease-free survival and overall survival (OS) rates (22). In addition, chemotherapy followed by local radiotherapy, compared with chemotherapy alone, demonstrated improved event-free survival (EFS) results (23). A previous study reported 5-year survival, EFS and OS rates of 94, 84 and 91%, respectively (24). However, ~50% of DLBCL patients are unresponsive to the standard chemotherapy or suffer disease relapse (19,20). Patients with refractory NHL have limited treatment options and poor prognosis. Hou *et al* (25) reported that GDP with or without rituximab was effective in patients with refractory or relapsed aggressive B cell NHL. The overall response rate of patients with recurrent history or patients with refractory aggressive histology B cell NHL is 49–72% (25–27). In addition, 28% of patients suffered grade III–IV neutropenia and 40% of patients suffered thrombocytopenia (25–27). Aribi *et al* (26) reported that the 3-year progression-free and EFS rates were 20.5% (range, 16.3–24%) and 19.7% (range, 15.9–23.5%) for the GPD regimen, respectively. These results indicated that GDP with or without rituximab may be a promising treatment option for refractory DLBCL. However, in the present study, the patient received a modified BCOAD regimen, which had not been previously reported; the results demonstrated that the treatment was effective in this patient and to date the patient has remained stable and healthy.

In conclusion, primary skeletal muscular DLBCL is a rare lesion that frequently occurs among elderly adults. Imaging can be used to reveal the location and the size of the mass. Biopsy or surgery are the key to the diagnosis of DLBCL. Selection of the appropriate treatment regimen is challenging and requires further investigation.
